# Patient-centered communication and shared decision making to reduce HbA1c levels of patients with poorly controlled type 2 diabetes mellitus - results of the cluster-randomized controlled DEBATE trial

**DOI:** 10.1186/s12875-019-0977-9

**Published:** 2019-06-25

**Authors:** Anja Wollny, Attila Altiner, Anne Daubmann, Eva Drewelow, Christian Helbig, Susanne Löscher, Michael Pentzek, Sara Santos, Karl Wegscheider, Stefan Wilm, Christin Löffler

**Affiliations:** 1 Institute of General Practice, Rostock University Medical Center, Doberaner Str. 142, 18057 Rostock, Germany; 2 Institute of Medical Biometry and Epidemiology, Medical Center Hamburg-Eppendorf, Martinistraße 52, 20246 Hamburg, Germany; 3 Institute of General Practice, Medical Faculty, Heinrich-Heine University Düsseldorf, Moorenstr. 5, 40225 Düsseldorf, Germany

**Keywords:** Diabetes mellitus type 2, Physician-patient relations, Decision making, Quality of life, Health communication, Health services research

## Abstract

**Background:**

Does an intervention designed to foster patient-centered communication and shared decision making among GPs and their patients with poorly controlled type 2 diabetes mellitus reduce the level of HbA1c.

**Methods:**

The DEBATE trial is a cluster-randomized controlled trial conducted in German primary care and including patients with type 2 diabetes mellitus having an HbA1c level of 8.0% (64 mmol/mol) or above at the time of recruitment. Data was measured before intervention (baseline, T0), 6–8 months (T1), 12–14 months (T2), 18–20 months (T3), and 24–26 months (T4) after baseline. Main outcome measure is the level of HbA1c.

**Results:**

In both, the intervention and the control group the decline of the HbA1c level from T0 to T4 was statistically significant (− 0.67% (95% CI: − 0.80,-0.54%; *p* < 0.0001) and − 0.64% (95% CI: − 0.78, − 0.51%; *p* < 0.0001), respectively). However, there was no statistically significant difference between both groups.

**Conclusions:**

Although the DEBATE trial was not able to confirm effectiveness of the intervention tested compared to care as usual, the results suggest that patients with poorly controlled type 2 diabetes are able to improve their blood glucose levels. This finding may encourage physicians to stay on task to regularly approach this cohort of patients.

**Trial registration:**

The trial was registered at ISRCTN registry under the reference ISRCTN70713571.

**Electronic supplementary material:**

The online version of this article (10.1186/s12875-019-0977-9) contains supplementary material, which is available to authorized users.

## Background

Since 1980 the global burden of diabetes mellitus type 2 is rising continuously. In Europe alone, 60 million people are affected by diabetes. This accounts for about 10% of adults aged 25 years and over [1]. Despite generally high availability of primary and specialized ambulatory care as well as the implementation of Disease Management Programs, 15–30% of all German patients with type 2 diabetes mellitus continue to have poorly controlled blood glucose levels, that is HbA1c levels of 8.0% (64 mmol/mol) or above [2]. A number of patient related factors have been found to impact on patients’ ability to adhere to diabetes treatment schemes including social support, self-confidence in managing diabetes, the availability of psycho-social resources and health literacy [3–6]. A qualitative study of German general practitioners (GPs) found GPs perceive that patients with poorly controlled blood glucose levels are less likely to see their doctor regularly or take the suggested dose of prescribed drugs and are unmotivated to making changes to diet, physical activity and smoking behavior [7].

Different interventions have been designed and tested to improve the health of patients living with type 2 diabetes. Research has shown that educational interventions relying merely on the provision of - mostly written - information have little effect on patient-related outcomes [8, 9]. Also, interventions that aim to improve patient adherence did not show any effects [10]. By contrast, quality of doctor-patient communication and patient satisfaction with primary health care influence health outcomes [11, 12]. Patient-centered behaviors have been found to positively affect patient’s ability to be an active participant in the consultation [13]. In addition, qualitative research underpins the importance of patient-centeredness for patients with type 2 diabetes [2].

This study aimed at evaluating whether an intervention that fosters patient-centered communication and shared decision making among GPs and their patients with poorly controlled type 2 diabetes mellitus is able to reduce the level of HbA1c in a clinically relevant way. We referred to patient-centered communication as a communication style that aims at eliciting the patient’s agenda using narrative, open-ended questions. We defined shared decision making as helping patients to explore their preferences and to make decisions in medical encounter.

## Methods

### Trial design

The DEBATE trial was a cluster-randomized controlled trial testing the hypothesis whether patient-centered communication and shared decision making are able to reduce the level of HbA1c by 0.5% between intervention and control group. Data was measured before randomization (baseline, T0), 6–8 months (T1), 12–14 months (T2), 18–20 months (T3), and 24–26 months (T4) after baseline.

### Recruitment, eligibility criteria, and sampling procedure

Participating GPs were recruited through registers of the regional Associations of Statutory Health Insurance Physicians of the German regions Mecklenburg-Western Pomerania and North Rhine-Westphalia. Both associations provided lists of GPs working in both regions of recruitment. GPs were contacted in waves until the planned number of participants was reached. It was anticipated that each study center, Rostock (Mecklenburg Western Pomerania), Witten (North Rhine-Westphalia), and Düsseldorf (North Rhine-Westphalia) would recruit 20 GPs, resulting in a total of 60 GPs.

Within each participating GP practice, a list of eligible patients was compiled electronically before randomization. Patients were eligible when they fulfilled the following inclusion criteria: being affected by type 2 diabetes mellitus, having an HbA1c level of 8.0% or above (64 mmol/mol) in the 3 months before recruitment, being able to provide informed consent, and having sufficient German language skills. Exclusion criterion was the presence of severe comorbidities resulting in an assumed life expectancy of less than 24 months. A total of 13 patients with poorly controlled diabetes mellitus type 2 were to be recruited from each practice. For practices with more than 13 eligible patients a random sample was created and GPs approached these eligible patients until the target number of 13 patients was reached. As most GPs were not able to identify 13 eligible patients a higher number of GPs was recruited. Participating patients were asked to give informed consent before participating in the study.

### Intervention

The intervention concept was based on a previous qualitative study with German general practitioners and their patients with poorly controlled type 2 diabetes. Findings suggested that GPs perceive themselves as ‘experts’ but describe some of their patients as in denial or refusing to follow advice. Given this gridlocked role pattern, GPs tended to give up hope for improvement and became resigned to the situation [14]. Based on this an intervention was developed that encouraged patient-centered communication and shared decision making. First, in order to enhance patient-centered communication specially trained GPs (peers) visited participating GPs in their practice to sensitize them for patients with poorly controlled type 2 diabetes. GPs were encouraged to address patients’ concepts of illness and to (re-)evaluate their patients’ views, attitudes and behaviors by using patient-centered communication. Second, an electronic decision-aid (https://www.arriba-hausarzt.de/) was provided to GPs to increase shared decision making. Based on HbA1c levels and associated risk factors the decision-aid visualized the probability of experiencing macro vascular events [15, 16]. Also, the impact of therapeutic options (such as oral medication or insulin therapy) and lifestyle changes on cardiovascular events were demonstrated and used for shared decision making. Out of 53 GPs randomized into intervention, 47 received peer-visits after baseline data collection (component 1). Six GPs were not available for the visit and received written information material and a phone call. Additionally, all GPs were offered the possibility to participate in a workshop on patient-centered communication. Altogether, 10 GPs from the intervention group took up the offer (component 2). Additional file 2: Table S6 and Additional file 3: Table S7 in the supplementary material provide a detailed description of both intervention components.

To enhance intervention fidelity, peers wrote a memo right after visiting participating GPs in their practice. The memo contained information on the following issues: where the visit took place, disrupting factors before, during and after the visit (calls, long waiting period before the visit, discontinuation if the visit etc.), in what mood was the GP - was he interested and motivated, how did the peer experience the visit (mood, associations, feelings), overall impression of the conversation, overall conclusion of the details of the conversation (ideas, critique, doubts of the GP), further observations/ comments referring to the project. However, we did not audio-tape or observe GPs actual consultations with their patients.

### Control

GPs of the control group did not participate in any intervention. They provided care as usual for their patients. In Germany, for patients with type 2 diabetes mellitus care as usual includes, for instance, measuring levels of HbA1c and consulting the GP two to four times a year.

### Primary and secondary outcomes

Primary endpoint was the HbA1c level at the time of the follow-up examinations. The level was delivered to the study team by practice staff as obtained in the patient record. Secondary endpoints included patients’ quality of life (BÄK questionnaire [2], EQ-5D [17], PAID [18]), shared decision making (SDM-Q-9 [19]) and patient-centeredness (PACIC-D [20]). Study team members collected data by telephone at the different points of measurement. Both, cardiovascular risk prognosis and prescribed drugs were secondary outcomes, too. Detailed information on data collection procedures were published elsewhere [21]. Also, results of secondary outcomes will be published elsewhere.

### Sample size

Assuming a residual standard deviation of 0.9, a case number of 2 × 143 (= 286) patients was calculated to realize a randomized study on the patient-side to show a difference in the HbA1c of 0.5% with a statistical power of 80%. Due to the design of the study (cluster-randomized study with an intervention on the GPs-side) the resulting cluster-effects had to be taken into account. The design factor was set at 1.9 assuming an ICC of 0.1 and an average cluster-size of 10. Assuming an average dropout rate of approx. 20%, it was calculated to recruit at least 54 GPs, who treated 13 patients each in order to assure no less than 10 eligible patients per GP [22]. Thus, the recruitment of 60 practices was required assuming a practice drop-out rate of 10%. In order to achieve the calculated sample size (*n* = 780 patients) the study was designed as a multi-centered study.

### Stopping rules

Within the DEBATE trial stopping rules were not defined.

### Randomization

Randomization was performed in clusters with one GP and his/her respective patients being one cluster. In Germany most GP practices are single handed. In order to prevent contamination, GPs of group practices were considered as one cluster. Within each study center unrestricted randomization was used to allocate the intervention to GPs and their selected patients. Randomization was performed by statisticians who were not involved in study procedures and realization.

### Allocation concealment mechanism

At study inclusion GPs were not informed about the implementation of a peer-visit among physicians of the intervention group. Participating GPs, therefore, were not aware of group allocation when recruiting their patients. Also, baseline data collection was performed before randomization.

### Blinding

Patients, GPs and scientists were not explicitly blinded. However, study nurses performing data collection (questionnaires) were not aware of group allocation and patients were not informed about the group they belong to.

### Statistical methods

Groupwise and total descriptive statistics including means, standard deviations or absolute and relative frequencies were calculated for all randomly assigned patients (intention-to-treat (ITT) population). The primary analysis was performed in the ITT-population. A longitudinal mixed model was applied to study the dependence of HbA1c changes from baseline from the study intervention and several covariates, with practice and patient as random effects, and follow-up visits as repeated measurements without restriction of the covariance matrix. Baseline HbA1c-value was added to the model for variance reduction. Recruiting center and time as fixed effects were included for control of potential biases. As potential covariates, marital status, age at diagnosis, number of persons in the household, and cardiovascular risk were considered on patient level as well as averaged on practice level, complemented by physician’s gender. The final set of covariates was to be selected by likelihood ratio based forward selection. Tentatively, the time by group interaction was to be tested; if the interaction was significant, the interaction was to be included in the model. If the interaction was not significant, the interaction was not to be included in the model. The coefficient test, comparing the adjusted HbA1c-values between the randomized groups, was performed using the direct maximum likelihood as the statistical estimation procedure, which results in unbiased estimators under the missing-at-random-assumption. The analysis of the primary outcome was repeated in the per protocol (PP) population. In the sensitivity analyses of the primary outcome, the missing values in the ITT population were replaced by last observation carried forward, multiple imputations, hotdeck imputation and a propensity score approach. Adjusted means with 95% confidence intervals and *p* values were reported. The significance level was set at two-sided α-level of 0.05. All analyses were conducted with SAS 9.4.

## Results

### Recruitment and participant flow

Over the period of 24 months, between 08/2011 and 07/2013, 833 patients of 108 GPs were recruited into the study. In the sample, all GPs worked in single-handed practices or - in case of group practices - were the only GP of that practice taking part in the trial. Thus, in this study one GP was equivalent with one practice. Information on the total number of patients approached for study participation has not been collected.

**Fig. 1:**
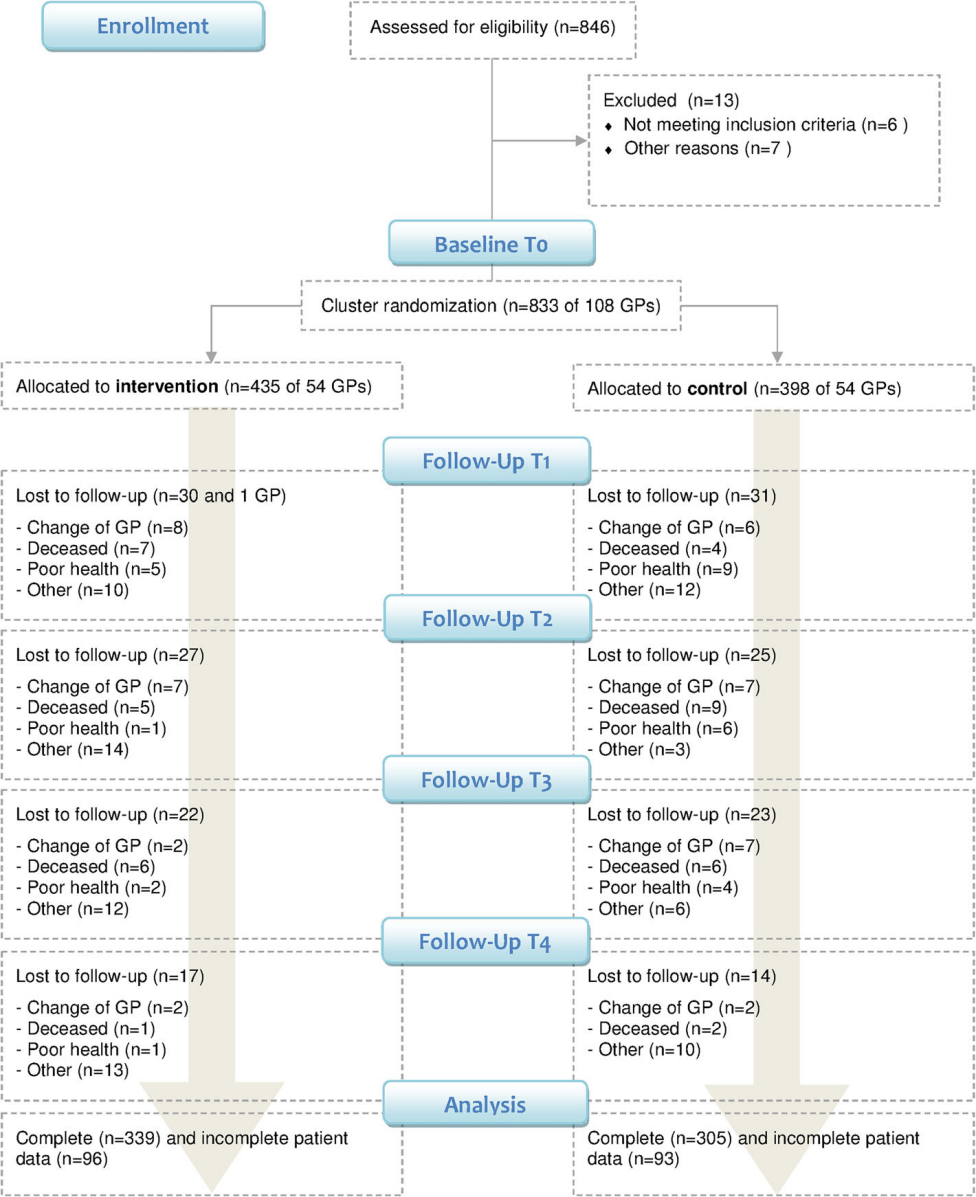
Flow chart of the DEBATE trial

Fify-four GPs with 435 of their patients were randomized to the intervention group, and 54 GPs with 398 patients were randomized to the control group. One GP of the intervention group left the study after T1. At T4, a total of 644 patients remained in the study. Patient drop-out rates were 22.1% in the intervention group and 23.4% in the control group. Most patient drop-outs were related to a change of GP, poor health or death of patients. Other reasons included patients’ loss of interest in the trial and failed attempts to re-contact trial participants. See Fig. 1 for the detailed flow of participants.

### Baseline data

Basic socio-demographic and health data of participating patients is provided in Table 1. With regard to socio-demographic characteristics there were no considerable differences between groups.

**Table 1:** Patient baseline data

	Intervention		Control		Total	
	N	%	N	%	N	%
Number of patients	435	52.2	398	47.8	833	100.0
Sex						
Male	241	55.4	212	53.3	453	54.4
Female	194	44.6	186	46.7	380	45.6
Age (median)	65.9		65.8		65.9	
Marital status^a^						
Single	46	10.6	41	10.3	87	10.5
Married	273	62.8	229	57.7	502	60.3
Divorced	30	6.9	52	13.1	82	9.9
Widowed	86	19.8	75	18.9	161	19.4
Living with a partner^b^						
Yes	291	67.1	252	63.6	543	65.4
No	143	32.9	144	36.4	287	34.6
Year of diagnosis (mean)	1999		2001		2000	

Notes: ^a^ one missing value, ^b^ three missing values

### Numbers analyzed

Data on HbA1c levels at T0 was available for 435 patients of the intervention group, and for 398 patients of the control group. To deal with loss to follow-up and missing data, we performed sensitivity analyses. Imputations for missing data did not alter results. See Additional file 1 Table S5.

### Outcomes and estimations

**Fig. 2:**
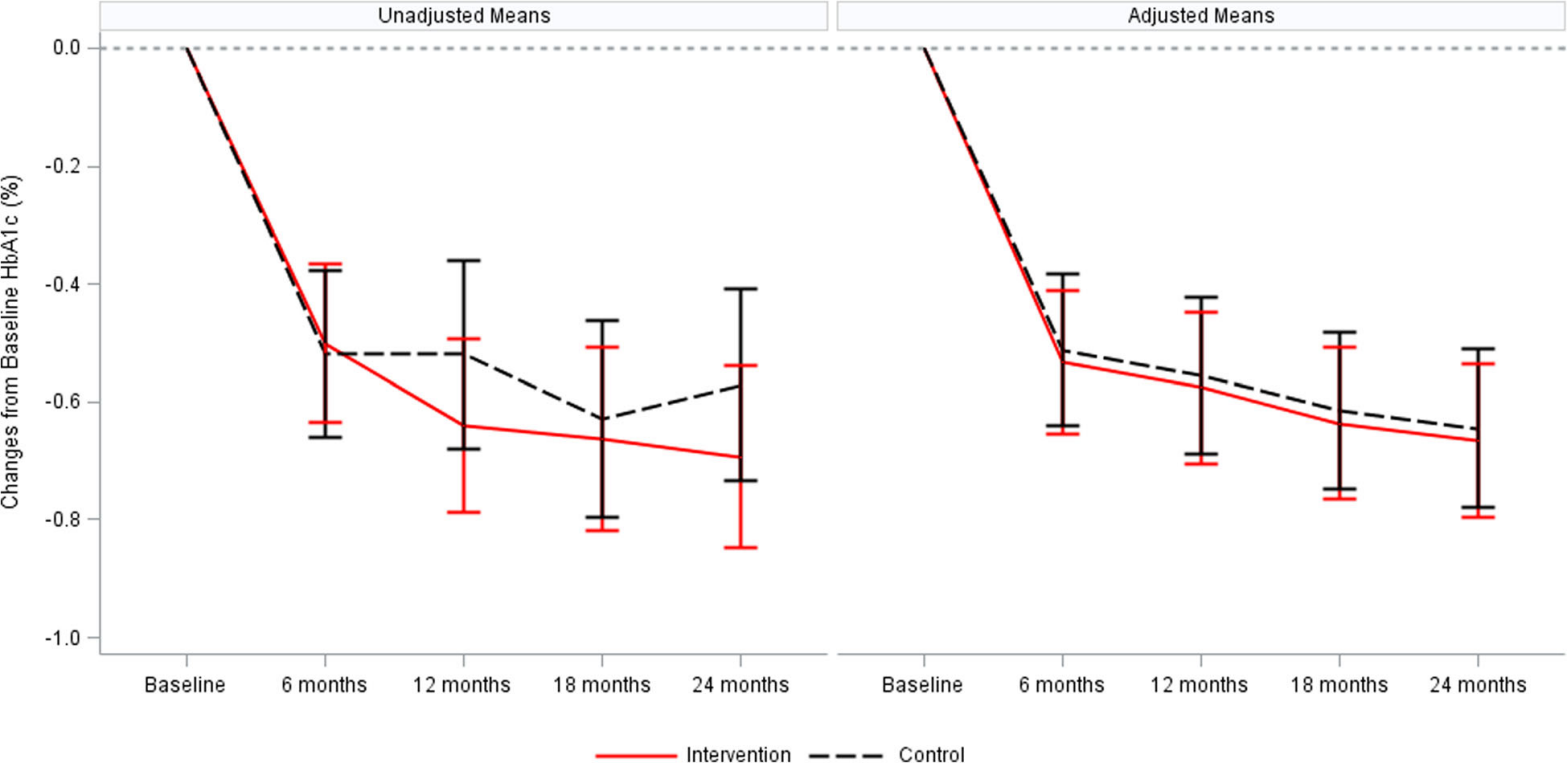
Unadjusted and adjusted mean changes of the HbA1c levels from baseline to all follow-ups by study group

At baseline, in the intervention group the unadjusted mean HbA1c level was 8.99% (75 mmol/mol) compared to 8.89% (74 mmol/mol) in the control group. Until T4, we observed a decline of the level in both study groups, reaching 8.21% (66 mmol/mol) in the intervention group and 8.34% (68 mmol/mol) in the control group. Based on this data we calculated mean changes of the HbA1c levels at each point of measurement compared to baseline and adjusted for the effects outlined in the statistical methods section above. In the final set of covariates, selected by likelihood ratio based forward selection, only age at diagnosis on patient level was chosen as an additional covariate. The time courses of HbA1c of the two groups were not significantly different. Mean changes of the HbA1c levels relative to baseline ranged between − 0.53% (95% CI: − 0.66, − 0.41) at T1 and − 0.67% (95% CI: − 0.80, − 0.54) at T4 in the intervention group, and between − 0.51% (95% CI: − 0.64, − 0.38) at T1 and − 0.64% (95% CI: − 0.78, − 0.51) at T4 in the control group. Unadjusted and adjusted mean changes of the HbA1c level are plotted in Fig. 2. Within each study group the decline of the HbA1c level from T0 to T4 was statistically significant (*p* < 0.0001). However, there was no statistically significant difference between both groups (see Table 2 and Fig. 2). Results of the per protocol analysis confirmed this finding (see Additional file 4:Table S8).

**Table 2:** Changes of the HbA1c level from baseline over all follow-ups among groups, between group differences, and interaction between group and time

		Intervention group							Control group						Between group differences				Interaction between
		Change from baseline							Change from baseline						Intervention group - Control group				group and time
	N	Mean	SD	Adjusted Mean	95% CI		*p*-Value	N	Mean	SD	Adjusted Mean	95% CI		*p*-Value	Adjusted Mean	95% CI		*p*-Value	*p* Value
**HbA1c**																			0.1538
Baseline	435	8.99	1.3					398	8.89	1.2									
6 months follow up	404	8.48	1.4	−0.53	−0.66	− 0.41	<.0001	366	8.39	1.4	− 0.51	− 0.64	− 0.38	<.0001	− 0.02	− 0.19	0.14	0.7985	
12 months follow up	376	8.32	1.4	−0.58	− 0.70	− 0.45	<.0001	339	8.40	1.4	−0.55	−0.69	− 0.42	<.0001					
18 months follow up	353	8.27	1.4	−0.64	−0.77	−0.51	<.0001	319	8.30	1.4	−0.62	−0.75	− 0.48	<.0001					
24 months follow up	338	8.21	1.4	−0.67	−0.80	−0.54	<.0001	305	8.34	1.4	−0.64	−0.78	− 0.51	<.0001					

### Ancillary analyses

To understand the extent to which changes in medications had an impact on the observed declines of the HbA1c levels among study groups, we assessed diabetes medications. First, we applied a model that used the sum of all diabetes medications as an outcome. This model showed that mean number of diabetes medications rose among both study groups: In the intervention group the mean number changed from 1.98 drugs at baseline to 2.14 drugs at T4. In the control group, we observed an increase from 2.02 drugs to 2.25 drugs over the same time period. Again, within each study group the observed changes were statistically significant (*p* < 0.001), whereas there was no significant difference between the groups (see Table 3).

**Table 3:** Changes of medication from baseline to 12 and 24 months follow-up among groups, between group differences, and interaction between group and time

		Intervention group							Control group						Between group differences			
		Change from baseline							Change from baseline						Intervention group - Control group			
Outcome variables	N	Mean	SD	Adjusted Mean	95% CI		p Value	N	Mean	SD	Adjusted Mean	95% CI		*p* Value	Adjusted Mean	95% CI		*p* Value
Sum of all diabetes medication																		
Baseline	435	1.98	0.8					398	2.02	0.8								
12 months follow up	378	2.09	0.9	0.05	−0.03	0.12	0.2170	342	2.11	0.9	0.09	0.01	0.17	0.0274	−0.04	−0.14	0.06	0.4316
24 months follow up	339	2.14	0.9	0.14	0.06	0.22	0.0006	305	2.25	0.9	0.18	0.10	0.27	<.0001				
Insulin																		
Baseline	435	0.97	0.9					398	0.97	0.9								
12 months follow up	378	1.08	0.9	0.09	0.03	0.15	0.0021	342	1.13	0.8	0.14	0.08	0.20	<.0001	−0.05	−0.12	0.03	0.2293
24 months follow up	339	1.17	0.9	0.20	0.14	0.26	<.0001	305	1.22	0.9	0.25	0.18	0.31	<.0001				
Sulfonylurea																		
Baseline	435	0.19	0.4					398	0.21	0.4								
12 months follow up	378	0.20	0.4	−0.00	−0.04	0.03	0.8920	342	0.17	0.4	−0.02	−0.05	0.01	0.2591	0.02	−0.03	0.06	0.4506
24 months follow up	339	0.16	0.4	−0.04	− 0.08	−0.01	0.0195	305	0.15	0.4	−0.06	−0.10	− 0.02	0.0014				
Other diabetes medication																		
Baseline	435	0.01	0.1					398	0.01	0.1								
12 months follow up	378	0.02	0.1	0.01	−0.01	0.03	0.4477	342	0.03	0.2	0.01	−0.01	0.03	0.1829	−0.01	−0.03	0.02	0.6704
24 months follow up	339	0.05	0.2	0.04	0.02	0.07	0.0003	305	0.07	0.2	0.05	0.03	0.07	<.0001				

In a second step, we divided diabetes medications into the five medication groups insulin, metformin, incretin, sulfonylurea and other diabetes medications, and analyzed them separately. We observed statistically significant changes for insulin, sulfonylurea and other diabetes medications. Increases within each study group were statistically significant for insulin (*p* < 0.0001), whereas there was no significant difference between the groups. Statistically significant decreases were found for sulfonylurea and other diabetes medication. Again, there was no statistically significant difference between the groups (see Table 3).

In addition, we analyzed patients with improved HbA1c levels of more than 0.2% (e.g. improvement from 8.4 to 8.2%) among both study groups over time and examined the proportion of patients receiving less, the same or more medications. Proportions differed somewhat between study groups (see Table 4). However, logistic regression models with changes in medications as response variable did not show any statistically significant results (models not shown).

**Table 4:** Changes of medication from baseline to 24 months follow-up among patients with improvements of the HbA1c level of more than 0.2%, by group

		Group						Total		
		Intervention			Control					
		Diabetes Medication			Diabetes Medication			Diabetes Medication		
		Less	No change	More	Less	No change	More	Less	No change	More
No improvement	Numbers	38	156	71	40	116	68	78	272	139
	Percentages	14%	59%	27%	18%	52%	30%	16%	56%	28%
Improvement	Numbers	14	35	24	8	47	26	22	82	50
	Percentages	19%	48%	33%	10%	58%	32%	14%	53%	32%

### Secondary outcomes

Secondary outcomes will be reported in detail elsewhere. However, we found that subjective shared decision making decreased in both groups during the course of the study. The two groups were not significantly different. Patient-centeredness was less straightforward: values increased in both groups, but the increase was not statistically significant, nor was the difference between the groups. Subjective shared decision making was measured among patients using the SDM-Q-9 [19] questionnaire. For measuring patient-centeredness among patients we used the PACIC-D [20] questionnaire. Information were collected at all times of measurement from T0 to T4 and independent from the fact whether patients made a decision or not, or had a consultation.

### Harms

Within the DEBATE trial outcomes such as the interaction of medications, cardiovascular events, or death of patients were not systematically assessed. Since we tested routine care against routine care and an intervention to promote patient-centered communication during a doctor-patient consultation, no harms were expected.

## Discussion

### Summary of findings

The DEBATE trial tested an intervention to promote patient-centered communication and shared decision making in consultations between GPs and their patients with poorly controlled diabetes mellitus type 2 and its effect on the level of HbA1c. The decline of the HbA1c level from T0 to T4 was statistically significant in both intervention and control group. However, there was no statistically significant difference between the groups. We additionally analysed changes in diabetes medications in order to investigate possible effects on levels of HbA1c. Within each study group the mean number of diabetes medications increased. Whereas this increase was statistically significant for each group, again there was no significant difference between the groups. As the individual analysis showed, the same was true for insulin.

### Interpretation in the context of existing literature

The decline of HbA1c levels in both study groups prompted the question whether and to what extent the observed changes might be ‘regression towards the mean’, i.e. natural reductions in patients who were selected for their maximum HbA1c. Several factors precluded us from calculating the size of this effect, e.g. the variability of the HbA1c itself, the possible inclusion of patients with a one-time high level of blood glucose, therapy effects, or the effect of ageing on HbA1c levels. However, data of the German Disease Management Program Diabetes Type 2 from the region North Rhine-Westphalia is available and gives some information on the potential size of the effect. The data shows, that among patients roughly comparable with those in our trial as far as blood glucose levels are concerned, over a period of 2 years HbA1c levels decreased on average by 0.37% [23]. It finally remains open to what extent regression to the mean impacted our findings.

In the past there has been evidence to support the positive impact on patient outcomes from GP training in communication and patient-centered behaviours [11–13]. In 2009, Zolnierek and Dematteo published a meta-analysis on more than 1 hundred studies and showed that training physicians to improve their communication with patients enhances patients’ adherence [24]. However, qualitative interviews conducted as part of process evaluation at the end of the DEBATE trial showed that GPs were only marginally able to recall the content of the peer visits. In retrospect, we recognize in fact discrepancies between the very intensive follow-up on the one side and the comparably surface delivery of the intervention on the other side. Although the trial might have enhanced overall awareness for poorly controlled type 2 diabetes among both, patients and physicians, it seems unlikely that it had sustainable impact on GPs counseling behavior. A different and more continuous way of intervention delivery might have been more appropriate.

With its focus on patient centeredness and shared decision making the DEBATE trial adds to the growing body of research on these new principles in health care. A recently updated Cochrane systematic review analyzed 87 studies to investigate whether activities to increase shared decision making by healthcare professionals are effective or not. The review, however, fails to provide a clear cut answer: As certainty of evidence of included studies was low or very low, it remains uncertain whether interventions to increase shared decision making are effective [25]. It is actually unclear how complex cultural changes such as the broad social movement for greater patient autonomy can be adequately measured [26]. Elwyn and colleagues propose a broader conceptualization and measurement of shared decision making that includes a set of proximal, distal, and distant consequences of changes towards shared decision making that act at different levels (the individual level, the group level, the organizational level, and the healthcare system) [27].

### Strengths and limitations

Systematic research on the management of diabetes mellitus in primary care criticizes inadequate time of follow-up in trials as well as high drop-out rates and poor number of participants [28–30]. With a follow-up period of 24 months and a total of five points of measurements, the DEBATE trial was adequate to identify potential short-term and medium-term effects. Also, drop-out rates among intervention and control group were within the limits of previous estimations. Furthermore, participating patients were sampled from the entire eligible patient base of GPs, a procedure minimizing selection bias.

However, given the intensive follow-up of patients, it seems to be plausible that the study had a sensitizing influence on patients of both study groups: The study methodology involved patients in both the intervention and control groups being contacted by researchers five times over the course of the study to complete a comprehensive list of questionnaires addressing well-being, personal goal setting, and patient empowerment. Each call took about 30 min. These follow-up telephone calls to collect patient outcomes may have acted as an intervention in itself and led to improvements in HbA1c levels.

Further, as we did not collect information on patients not willing to participate in the trial, we cannot estimate the potential impact of selection bias on the study.

Also, for the measurement of long-term effects the focus on micro and macro vascular events as outcome measures is desirable. However, given time and cost constraints this procedure was hardly feasible. We therefore decided for HbA1c levels as proxy outcome being aware of ongoing discussions on its appropriateness. Also, the inability to quantify the regression to the mean effect within the trial limits the impact of the study. Collecting data of all patients with poorly controlled diabetes type 2 among participating GPs might have allowed to calculate this effect and to compare it with both study groups. Last but not least, to empower patients, elements of the interventions should have had focused on patients and not only on GPs.

### Clinical impact

The DEBATE trial showed that patients with poorly controlled type 2 diabetes were able to improve their blood glucose levels. The intensive and individual follow-up of patients, which went far beyond conventional disease management programs, might explain part of the effect. This may encourage physicians to stay on task to regularly approach this vulnerable and hard-to-reach group of patients.

### Future research

Future studies should continue investigating the effectiveness and efficiency of interventions to improve management of diabetes mellitus type 2, especially among patients suffering from poorly controlled blood glucose levels. Qualitative research is able to explore the fit of interventional concepts among different groups of patients, settings and countries. This is important, as tailored concepts tend to be more successful in changing health behavior.

## Conclusions

The DEBATE trial was not able to confirm effectiveness of the intervention tested compared to care as usual. In the intervention and control group patients with poorly controlled type 2 diabetes were able to improve their blood glucose levels. This finding may encourage physicians to stay on task to regularly approach this vulnerable and hard-to-reach group of patients.

## Additional files

Additional file 1: **Table S5.** Sensitivity analysis (DOCX 22 kb)

Additional file 2: **Table S6.** Intervention description of component 1: Outreach educational peer visit (according to TIDieR) (DOCX 14 kb)

Additional file 3: **Table S7.** Intervention description of component 2: Additional training on patient communication skills for GPs - optional (according to TIDieR). (DOCX 15 kb)

Additional file 4: **Table S8.** Changes of the HbA1c level from baseline over all follow-ups among groups, between group differences, and interaction between group and time; per protocol analysis. (DOCX 17 kb)

## Data Availability

The datasets generated and analysed during the current study are available from the corresponding author on reasonable request.

## Abbreviations

GP: General Practitioner
ITT: Intention-to-treat
PP: Per Protocol
