# Supplementary material for: Patient-centered communication and shared decision making to reduce HbA1c levels of patients with poorly controlled type 2 diabetes mellitus - results of the cluster-randomized controlled DEBATE trial
Source: BMC Fam Pract. 2019 Jun 25;20:87. doi: 10.1186/s12875-019-0977-9 (PMC6593484; doi:10.1186/s12875-019-0977-9)
Supplement: Supplementary file 1 — Table S5. Sensitivity analysis (DOCX 22 kb) [file 12875_2019_977_MOESM1_ESM.docx]

Table 5: Sensitivity analysis.

|  |  | **Intervention group** | | |  |  |  |  |  | **Control group** | | |  |  |  |  | **Between group differences** | | | |  | **Interaction between** |
| --- | --- | --- | --- | --- | --- | --- | --- | --- | --- | --- | --- | --- | --- | --- | --- | --- | --- | --- | --- | --- | --- | --- |
|  |  |  |  | Change from baseline | | | |  |  |  |  | Change from baseline | | |  |  | Intervention group - Control group | | | |  | **group and time** |
|  | N | Mean | SD | Adjusted Mean | 95% CI | | p-Value |  | N | Mean | SD | Adjusted Mean | 95% CI | | p-Value |  | Adjusted Mean | 95% CI | | p-Value |  | p Value |
| **HbA1c - Last observation carried forward** | | | |  |  |  |  |  |  |  |  |  |  |  |  |  |  |  |  |  |  | 0.1588 |
| Baseline | 435 | 8.99 | 1.3 |  |  |  |  |  | 398 | 8.89 | 1.2 |  |  |  |  |  |  |  |  |  |  |  |
| 6 months follow up | 435 | 8.52 | 1.4 | -0.48 | -0.59 | -0.36 | <.0001 |  | 398 | 8.41 | 1.4 | -0.45 | -0.57 | -0.33 | <.0001 |  | -0.03 | -0.18 | 0.13 | 0.7204 |  |  |
| 12 months follow up | 435 | 8.40 | 1.4 | -0.53 | -0.65 | -0.42 | <.0001 |  | 398 | 8.43 | 1.4 | -0.51 | -0.63 | -0.38 | <.0001 |  |  |  |  |  |  |  |
| 18 months follow up | 435 | 8.40 | 1.4 | -0.57 | -0.69 | -0.46 | <.0001 |  | 398 | 8.34 | 1.4 | -0.55 | -0.67 | -0.42 | <.0001 |  |  |  |  |  |  |  |
| 24 months follow up | 435 | 8.35 | 1.5 | -0.59 | -0.71 | -0.47 | <.0001 |  | 398 | 8.37 | 1.4 | -0.56 | -0.68 | -0.44 | <.0001 |  |  |  |  |  |  |  |
| **HbA1c - Hotdeck imputation** | |  |  |  |  |  |  |  |  |  |  |  |  |  |  |  |  |  |  |  |  | 0.3413 |
| Baseline | 435 | 8.99 | 1.3 |  |  |  |  |  | 398 | 8.89 | 1.2 |  |  |  |  |  |  |  |  |  |  |  |
| 6 months follow up | 435 | 8.39 | 1.6 | -0.61 | -0.74 | -0.47 | <.0001 |  | 398 | 8.33 | 1.6 | -0.55 | -0.69 | -0.40 | <.0001 |  | -0.06 | -0.24 | 0.12 | 0.5058 |  |  |
| 12 months follow up | 435 | 8.28 | 1.6 | -0.65 | -0.78 | -0.51 | <.0001 |  | 398 | 8.36 | 1.6 | -0.59 | -0.73 | -0.45 | <.0001 |  |  |  |  |  |  |  |
| 18 months follow up | 435 | 8.26 | 1.6 | -0.69 | -0.83 | -0.56 | <.0001 |  | 398 | 8.30 | 1.5 | -0.63 | -0.77 | -0.49 | <.0001 |  |  |  |  |  |  |  |
| 24 months follow up | 435 | 8.23 | 1.6 | -0.72 | -0.86 | -0.58 | <.0001 |  | 398 | 8.28 | 1.6 | -0.66 | -0.80 | -0.51 | <.0001 |  |  |  |  |  |  |  |
| **HbA1c - Multiple imputation** | |  |  |  |  |  |  |  |  |  |  |  |  |  |  |  |  |  |  |  |  | 0.1682 |
| Baseline | 435 | 8.99 | 1.3 |  |  |  |  |  | 398 | 8.89 | 1.2 |  |  |  |  |  |  |  |  |  |  |  |
| 6 months follow up | 435 | 8.48 | 1.5 | -0.53 | -0.65 | -0.42 | <.0001 |  | 398 | 8.40 | 1.5 | -0.45 | -0.57 | -0.33 | <.0001 |  | -0.07 | -0.23 | 0.07 | 0.2769 |  |  |
| 12 months follow up | 435 | 8.32 | 1.5 | -0.61 | -0.73 | -0.49 | <.0001 |  | 398 | 8.41 | 1.5 | -0.53 | -0.65 | -0.40 | <.0001 |  |  |  |  |  |  |  |
| 18 months follow up | 435 | 8.28 | 1.5 | -0.68 | -0.80 | -0.55 | <.0001 |  | 398 | 8.31 | 1.5 | -0.60 | -0.73 | -0.47 | <.0001 |  |  |  |  |  |  |  |
| 24 months follow up | 435 | 8.25 | 1.6 | -0.67 | -0.81 | -0.54 | <.0001 |  | 398 | 8.35 | 1.7 | -0.59 | -0.73 | -0.46 | <.0001 |  |  |  |  |  |  |  |
