# Supplementary material for: Patient-centered communication and shared decision making to reduce HbA1c levels of patients with poorly controlled type 2 diabetes mellitus - results of the cluster-randomized controlled DEBATE trial
Source: BMC Fam Pract. 2019 Jun 25;20:87. doi: 10.1186/s12875-019-0977-9 (PMC6593484; doi:10.1186/s12875-019-0977-9)
Supplement: Supplementary file 2 — Table S6. Intervention description of component 1: Outreach educational peer visit (according to TIDieR) (DOCX 14 kb) [file 12875_2019_977_MOESM2_ESM.docx]

Table 6: Intervention description of component 1: Outreach educational peer visit (according to TIDieR).

| 1 Short Name | Educational peer visit |
| --- | --- |
| 2 Goal and rationale | Improvement of doctor-patient communication and interaction between GP and patient, raising GPs awareness for patients with poorly controlled diabetes type 2, their individual agenda and concepts of illness and taking it into account in the process of shared decision making, putting the patient perspective more in the focus without overstraining both, doctor and patient. |
| 3 Materials | Oral input, computer-based decision-aid tool arriba-debate, discussion |
| 4 Procedures | Specially trained GPs visited participating GPs in their practice. During the visitation, specific problems/factors influencing the doctor-patient-communication and the treatment of patients with poorly controlled type 2 diabetes were collegially discussed with the GP (e.g. different ideas of therapy on GPs and patient's sides resulting in ineffective doctor-patient communication, lack of interest, resignation, frustration, anger). In addition, the peer GP introduced the basics of narrative based communication to the GP, gave individual feedback to patient cases the GP had experienced to be difficult. Additionally, during the visitation, the computer-based decision-aid tool arriba-debate was introduced to the GP. The tool visualises the patient the effect of possible changes of behaviour (e.g. smoking stop, exercise) and therapy (medication) on the individual risk of coronary heart disease under consideration of individual parameters (e.g. sex, age, blood pressure, cholesterol, blood glucose level). |
| 5 Providers of intervention | Trained general practitioners (peers) |
| 6 Mode of delivery | On site visit, oral presentation, introduction of the decision-aid tool and discussion |
| 7 Location | Visitation in the GP practice |
| 8 Frequency | Once after completing baseline between the 3rd quarter of 2012 and the 1st quarter of 2013; duration approximately 1-1.5h, total of 47 intervention practices received a peer visit |
| 9 Planned tailoring | No |
| 10 Fidelity enhancement | Memo written by peer |
