# Supplementary material for: Patient-centered communication and shared decision making to reduce HbA1c levels of patients with poorly controlled type 2 diabetes mellitus - results of the cluster-randomized controlled DEBATE trial
Source: BMC Fam Pract. 2019 Jun 25;20:87. doi: 10.1186/s12875-019-0977-9 (PMC6593484; doi:10.1186/s12875-019-0977-9)
Supplement: Supplementary file 3 — Table S7. Intervention description of component 2: Additional training on patient communication skills for GPs - optional (according to TIDieR). (DOCX 15 kb) [file 12875_2019_977_MOESM3_ESM.docx]

Table 7: Intervention description of component 2: Additional training on patient communication skills for GPs - optional (according to TIDieR).

| 1 Short Name | Additional training for GPs to promote patient-centred communication. |
| --- | --- |
| 2 Goal and rationale | The focus was on exploring individual patient expectation and concepts of illness and taking it into account in the process of shared decision making in patients with poorly controlled diabetes type 2. The educational objective was to carry out a convincing physician-patient communication and to overcome possible barriers existing in doctor-patient-communication. |
| 3 Materials | Theoretical input on narrative-based communication, group training on practical use of these skills, computer-based decision-aid arriba-debate |
| 4 Procedures | Theoretical background of narrative-based communication (incl. three-step-conversation) were introduced at the beginning of the training. In three group training sessions participants (max. 3-4) were asked to practically use these skills considering personal experiences and defaulted roles. The issues of the sequences differed, starting with a low-threshold one (e.g. vacation), followed by the experience of an in-acute disease (e.g. cold), ending with a practical oriented issue (e.g. GP as protagonist in the practice). Roles were changed after every session (narrator, asker, observer) to give all participants the opportunity to slip in each role. Subsequently, feedback about the practical implementation was given and discussion about transferability in daily routine was carried out.  Finally, the computer-based decision-aid tool arriba-debate and its use in daily routine in the GP-practice has been discussed. |
| 5 Providers of intervention | The training was performed by qualified scientific researchers of the study sites in Rostock, Düsseldorf and Witten. |
| 6 Mode of delivery | Single intervention  10 out of the 54 GPs in the intervention group of DEBATE |
| 7 Location | The training was performed in two of the study sites.  Total of five trainings with altogether 10 GPs were performed. |
| 8 Frequency | Each training lasted about 3 hours |
| 9 Planned tailoring | No |
| 10 Fidelity enhancement |  |
