# Supplementary material for: Patient-centered communication and shared decision making to reduce HbA1c levels of patients with poorly controlled type 2 diabetes mellitus - results of the cluster-randomized controlled DEBATE trial
Source: BMC Fam Pract. 2019 Jun 25;20:87. doi: 10.1186/s12875-019-0977-9 (PMC6593484; doi:10.1186/s12875-019-0977-9)
Supplement: Supplementary file 4 — Table S8. Changes of the HbA1c level from baseline over all follow-ups among groups, between group differences, and interaction between group and time; per protocol analysis. (DOCX 17 kb) [file 12875_2019_977_MOESM4_ESM.docx]

Table 8: Changes of the HbA1c level from baseline over all follow-ups among groups, between group differences, and interaction between group and time; per protocol analysis.

|  |  | **Intervention group** | | |  |  |  |  |  | **Control group** | | |  |  |  |  | **Between group differences** | | | |  | **Interaction between** |
| --- | --- | --- | --- | --- | --- | --- | --- | --- | --- | --- | --- | --- | --- | --- | --- | --- | --- | --- | --- | --- | --- | --- |
|  |  |  |  | Change from baseline | | | |  |  |  |  | Change from baseline | | |  |  | Intervention group - Control group | | | |  | **group and time** |
|  | N | Mean | SD | Adjusted Mean | 95% CI | | p Value |  | N | Mean | SD | Adjusted Mean | 95% CI | | p Value |  | Adjusted Mean | 95% CI | | p Value |  | p Value |
| **HbA1c** |  |  |  |  |  |  |  |  |  |  |  |  |  |  |  |  |  |  |  |  |  | 0.2023 |
| Baseline | 357 | 8.99 | 1.3 |  |  |  |  |  | 374 | 8.83 | 1.1 |  |  |  |  |  |  |  |  |  |  |  |
| 6 months follow up | 332 | 8.47 | 1.5 | -0.52 | -0.66 | -0.39 | <.0001 |  | 343 | 8.31 | 1.3 | -0.50 | -0.63 | -0.37 | <.0001 |  | -0.03 | -0.20 | 0.14 | 0.7473 |  |  |
| 12 months follow up | 310 | 8.30 | 1.5 | -0.56 | -0.70 | -0.42 | <.0001 |  | 316 | 8.37 | 1.4 | -0.53 | -0.67 | -0.40 | <.0001 |  |  |  |  |  |  |  |
| 18 months follow up | 294 | 8.23 | 1.4 | -0.63 | -0.76 | -0.49 | <.0001 |  | 297 | 8.28 | 1.4 | -0.60 | -0.73 | -0.46 | <.0001 |  |  |  |  |  |  |  |
| 24 months follow up | 283 | 8.18 | 1.5 | -0.65 | -0.79 | -0.51 | <.0001 |  | 286 | 8.27 | 1.3 | -0.63 | -0.76 | -0.49 | <.0001 |  |  |  |  |  |  |  |
